# The Search for Clinically Useful Biomarkers of Complex Disease: A Data Analysis Perspective

**DOI:** 10.3390/metabo9070126

**Published:** 2019-07-02

**Authors:** Elizabeth C. Considine

**Affiliations:** The Irish Centre for Fetal and Neonatal Translational Research (INFANT), Department of Obstetrics and Gynaecology, University College Cork, T12 YE02 Cork, Ireland; l.considine@ucc.ie

**Keywords:** complex disease, heterogeneous disease, biomarker discovery, data analysis, metabolomics, reproducibility, modeling, precision medicine

## Abstract

Unmet clinical diagnostic needs exist for many complex diseases, which it is hoped will be solved by the discovery of metabolomics biomarkers. However, as yet, no diagnostic tests based on metabolomics have yet been introduced to the clinic. This review is presented as a research perspective on how data analysis methods in metabolomics biomarker discovery may contribute to the failure of biomarker studies and suggests how such failures might be mitigated. The study design and data pretreatment steps are reviewed briefly in this context, and the actual data analysis step is examined more closely.

## 1. Introduction

Unmet clinical diagnostic needs exist for many complex diseases, which it is hoped will be solved by the discovery of metabolomics biomarkers. However, the molecular biomarker discovery effort and the field of precision medicine are currently experiencing skepticism and a loss of credibility due to high failure rates of the majority of proposed biomarkers in subsequent validation studies [1]. The United States Food and Drug Administration (FDA) currently only approves one to three new biomarkers a year, despite abundant literature on proposed novel molecular biomarkers [2]. There have been no metabolomics tests with FDA clearance since the emergence of modern metabolomics [3]. Putative biomarkers are failing at the initial verification and validation stages, and the “science of biomarker failures” has been described as ubiquitous, with less than 1% of published biomarkers entering clinical practice [4]. 

Clearly, there are problems in the biomarker discovery pipeline that are not conveying appropriate biomarker candidates for clinical application. Biomarker failure begins in discovery [5]. There are numerous opportunities for fatal flaws to accumulate from the early biomarker discovery phase (the preclinical exploratory phase) leading to the failure of a biomarker discovery project, including, but not limited to, poorly designed, underpowered studies that fail to take account of disease heterogeneity; false discoveries from biological variability or technically induced variability; inappropriate employment of data analysis methods and subsequent incorrect interpretation of findings, leading to over optimistic and misleading preliminary results; a lack of expert knowledge incorporated into the discovery phase; and promotion of discriminating, but not necessarily clinically actionable, candidate biomarkers [4]. 

For this review, the starting point of data analysis is deemed to be the metabolomics data matrix that exists after preprocessing (alignment, peak identification, batch correction, etc.) has been carried out. Such preprocessing methods are often platform dependent, and there are many open source software options for the data preprocessing step. A detailed list of open source software for metabolomics preprocessing (and indeed other aspects of metabolomics data analysis) has been compiled by Spicer et al. [6]. Obviously, there are challenges at the preprocessing stage of the pipeline, which will also affect subsequent analysis and successful biomarker discovery. For issues and challenges related to data preprocessing, the reader is directed to several comprehensive reviews in that area [7,8,9]. However, an examination of all the issues related to biomarker failures in the discovery phase is beyond the scope of this review, which is presented as a perspective on how data analysis methods in metabolomics may contribute to the failure of biomarker studies. The study design and data pretreatment steps are reviewed briefly in this context, and the actual data analysis step is examined more closely. 

## 2. Definition of a Complex Disease

First, however, we will define a complex disease. Most common complex diseases are categorised according to superficial, clinically observable features. At a molecular level these complex diseases are suspected to be polygenic, arising from the interplay of many genetic factors (and interactions with the environment). Some complex diseases could be heterogeneous, in which many different subtypes exist, themselves comprised of polygenic disorders, but not necessarily so, as they could comprise a homogenous group of diseases arising from the same polygenic architecture or gene set. Conversely, heterogeneous disorders could be complex (arising from many different polygenic variants), but also not necessarily so, as they could actually consist of a grouping, under an umbrella term, of a collection of phenotypically similar disorders, each arising from a single rare genetic variant. 

In the early stages of the Genome Wide Association Studies (GWAS) era, complex diseases were suspected to arise from many common variants [10]. However GWAS did not uncover the expected common variants that would explain polygenic inheritance but instead lead to the identification of many rare individually causal variants, indicating a high degree of genetic heterogeneity underlying common complex conditions [11,12]. Currently, the view of complex diseases is that so-called common disorders actually encompass many distinct genetic disorders. GWAS are not expected to reveal predictive biomarkers of disease, as most complex disease susceptibility variants have not shown a clear association with prognosis [13]. Metabolomics is the omics domain expected to overcome the limitations of GWAS as a diagnostic/predictive biomarker tool, as it represents the endpoint of the omics cascade and is the closest domain to the phenotype [13,14]. Also, particularly usefully for the identification of diagnostic biomarkers for complex diseases, depending on sample timing, samples in a metabolomics dataset may represent the convergence, in a common metabolic pathway, of many diverse etiological pathways (that arise from different genetic origins). This would cause the samples to exhibit the same phenotypic traits and have similar metabolomic profiles. However, no diagnostic tests based on metabolomics have yet been introduced to the clinic. Among the reasons for this is the failure of many preliminary findings from the exploratory phase to replicate in subsequent validation studies [15]. This failure of subsequent reproducibility in validation tests is part of the phenomenon of the reproducibility crisis.

The terms complex, multifactorial, and polygenic and even heterogeneous are, confusingly, often used interchangeably in the literature when describing disease [16,17]. This is because the underlying genetic architecture of these diseases is usually unknown. It is likely that most diseases that are known clinically as complex diseases (and their resultant datasets) are both complex and heterogeneous, i.e., a collection of phenotypically similar disorders each arising from varying polygenic architecture and possibly single rare gene variants. For the remainder or this paper, complex and/or heterogeneous disorders are referred to as CHD.

It would seem inevitable that a clear understanding and delineation of the nature of the molecular complexity of the disease (and the dataset) under study is essential to designing an appropriate study and data analysis plan. However, if a clear understanding of the molecular architecture is unknown (as is usually the case), an appreciation of this serious limitation and the potential hidden substructure in the data should be factored into the study design and the data analysis.

## 3. Study Design and the Biomarker Discovery Pipeline: Considerations for Complex Disease

Experimental design for a biomarker study describes the definition of the populations of interest, the selection of individuals to take part in the study, and the arrangement of the experimental material in space and time. Study design, therefore, is a subsection of experimental design that concerns itself with the selection of the individuals for the relevant phase of the biomarker discovery project. Here, we will briefly discuss issues related to study design for the preclinical exploratory phase of a CHD biomarker discovery project.

The preclinical exploratory phase has two goals: To identify leads for potentially useful biomarkers and to prioritise identified leads [18]. Study design decisions in the preclinical exploratory phase are dominated by bias, measurement, and inference concerns. However, the final study design is often a compromise between the idealised design and a number of logistical and ethical barriers surrounding the feasibility of recruitment and biological specimen collection [19]. Biases common in the discovery phase are selection bias, ascertainment of endpoint bias, inappropriate selection of controls, technically induced bias at sample acquisition and assaying stages, and bias introduced by statistical analysis. [20]. Less rigor is often employed in the discovery phase compared with the validation phase. For example, hospital based convenient samples are often used at the exploratory stage of the biomarker project, but these samples are often inherently biased [21]. Poor study design in the discovery phase without doubt contributes to biomarker failure, and discovery phase study design should follow rigorous principles to avoid bias [18,21,22]. 

Nested case control (NCC), a variant of the classic case control design where cases and controls are sampled from a well-defined cohort, is the recommended study design in biomarker discovery projects [23,24,25] as it is better able to handle the challenges of the effects of analytic batch, long term storage, and freeze-thaw cycles on biomarkers. It is also logistically simple, cost effective, and can be done with or without matching [26]. 

The PROspective-specimen collection, retrospective Blinded Evaluation (PROBE) study design is a type of NCC that was introduced by Pepe and colleagues in 2008 [25] for the biomarker validation stage, and its principles are now routinely adhered to in validation research. The PROBE design has since been recommended as equally important for the discovery phase to minimise inherent bias [21,27]. The main principles of the PROBE guidelines are 1. Consider the clinical endpoint early in study design. 2. Sample appropriately. 3. Collect specimens prospectively. 4. Select cases and controls retrospectively and randomly. 5. Carry out assay in a blinded fashion. Strict adherence to the PROBE design may not always be possible in the exploratory biomarker discovery phase, but it should be the goal.

PROBE authors also recommend an approach to sample size calculations that is similar to previous recommendations for sample size calculations for validation studies, except that the approach for the discovery phase addresses its unique challenge of filtering through many candidate biomarkers. The recommendations for the sample size calculations for the discovery phase require specifying—a definition for biomarker performance, the proportion of useful markers that the study should identify, and the tolerable number of useless markers among those identified. The PROBE approach allows for the selection of a panel of markers, where each marker meets a performance criterion that may be less than the clinical goal. The authors also draw attention to the fact that the statistical challenge of deriving combinations of biomarkers using classification or regression techniques (as is the norm in most metabolomics biomarker discovery studies) is “enormous” and that the enormity of the task is “widely underappreciated” [21].

Wallstrom et al. [28] suggest using a two stage design for biomarker discovery to deal with heterogeneity of disease. According to their pipeline, the first stage uses a portion of patients and controls to screen the full collection of biomarker candidates to eliminate implausible markers. In the second stage, the remaining candidates are screened using the remaining patients and controls. The advantage of this pipeline is that in the second stage, the number of candidates is sufficiently small that more detailed studies may be feasible. However, for heterogeneous disease, they do not recommend this approach for less than 100 cases and controls. An alternative approach to deal with heterogeneity includes a “phase zero” or pre-screen [4], which is described as an “intellectual process to identify plausible sets of markers” even before laboratory work starts. 

It has been proposed that the limitations imposed by the heterogeneity of datasets in biomarker discovery can be offset by increasing sample size [28]. Successful biomarker discovery for heterogeneous disease requires more than double the number of cases and controls than for homogeneous disease, and the probability of finding a true biomarker that exists in a heterogeneous dataset using 50 cases and 50 controls is only 15% [28]. Others have countered this, however, and suggest that homogeneity of sample groups is more important than sample size [29]. The latter point is echoed by Lemos et al., who state that.

From a scientific standpoint biomarker discovery should be ideally performed in relatively homogenous populations using narrowly defined endpoints that represent the most specific phenotypes possible. In contrast validation should be performed in diverse cohorts that better reflect the clinical circumstances in which biomarkers might be used [30].

If homogeneous discovery populations are not possible then large sample sizes are needed [28]. 

Matching is frequently employed in biomarker discovery to minimise confounding. However, matching on mediators, that are naturally connected to the outcome, will undermine the discovery process and can lead to biased estimates of prediction performance [31], a fact which is usually not appreciated in metabolomics literature. Matching makes controls more similar to cases on all correlates of the matching factor [32], leading to cases and controls having similar biomarker profiles. The ramification of this is that the performance of a valid biomarker will appear to be deflated as true differences are obscured. Anti-matching [33] is a matching technique where cases are matched to controls counter to known risk factors of the disease, i.e., for a disease for which age and ethnicity are known to be associated with disease, then a control is selected for a case with a deliberately different age category or ethnicity category. In this way, a known pattern of bias is introduced into the data, which leads to the observed specificity of a true biomarker being higher in discovery studies. 

Matching in biomarker discovery for CHD is a precarious issue as a particular exposure (variable) associated with disease is likely to only be dysregulated in a subgroup of cases. This implies that even for a variable/exposure of interest, a proportion of the case group that is not represented by that feature may be more like controls than the other cases for that particular feature. Overmatching is a risk in this situation, and biomarker discovery could be undermined. The anti-matching scheme may be particularly suitable for CHD datasets to reveal subtle, subgroup specific biomarkers, which are at risk of being obscured.

## 4. Data Pretreatment Step: Considerations for complex disease

Random and systemic bias and missing values are prevalent in (Mass Spectrometry) MS-based data due to biological, experimental and technical reasons [34]. Typical data pre-treatment methods, including filtering, normalization, scaling, and transformation are performed on MS datasets in an attempt to remove biases from the data. Imputation of missing values is carried out to provide a complete dataset for further analyses. 

Data pretreatment methods, and the order in which they are applied to the data, have been found to have a profound influence on the results of further data analysis [34,35]. Despite its importance, however, the data pretreatment step is largely overlooked in the reporting of biomarker discovery studies, indicating that its crucial role and influence on subsequent results are not fully appreciated [36].

At the earliest stage, before analysis starts, efforts should be made to reduce the dimensions of the dataset wherever possible, to enable true potential biomarkers to be revealed [37] and to address the curse of dimensionality [38]. Filtering at the outset can be performed to remove implausible features, that is, those features unlikely to be of clinical utility, thereby also reducing the complexity of the dataset. 

In a typical metabolomics dataset, each row represents a different sample and each column represents a different metabolite. Sample wise normalisation describes the row-wise operations carried out to reduce inter sample (typically inter person) variation, which is due to biological variation and, therefore, make each row comparable to each other. Normalisation methods can be either data driven or method driven. Data driven methods are used for untargeted (Liquid Chromatography Mass Spectrometry) LCMS data [39] and include normalization by sum or median normalization. Data driven methods depend upon certain assumptions, namely that a large amount of the metabolites in a sample stay constant and that only a small portion of the features will undergo significant experiment-related changes in expression.

Feature wise normalisation methods are column wise operations to adjust for the differences in abundances between the individual metabolites and, therefore, make each column comparable to each other. These include scaling, centering, and transformation. Scaling can use either a measure of the data dispersion or a size measure as a scaling factor. Heterogeneity in the form of hidden substructure in a disease dataset and in the form of the presence of features with dysregulation in a subset of samples can lead to scaling factors that introduce bias into the scaled dataset. Scaling by mean or standard deviation of controls only is one possible way of reducing bias, at least for features that are exhibiting stable expression in controls. Inappropriate normalisation methods can significantly impair data [40]. A comprehensive description and evaluation of pretreatment methods can be found in [41], where the authors conclude that different pretreatment methods emphasise different aspects of the data and that pretreatment methods should be selected while bearing in mind the biological question of interest, the properties of the dataset under investigation, and the data analysis method that will be used.

Missing values occur in LCMS data at rates of approximately 20% and affect up to 80% of variables [35]. How these missing values are dealt with or imputed massively influences the results of downstream analysis [35,42]. These missing values can be 1. missing completely at random (MCAR), i.e., missing due to not being measured correctly but actually being present in the sample, or 2. missing not at random (MNAR), i.e., abundance dependent missingness due to the abundance of the molecule falling below the instrument detection limit or due to the molecule simply not being present in the sample for a genuine biological reason. The typology MNAR/MCAR was coined by Rubin [43]. The majority of missing values in untargeted metabolomics data are expected to be MNAR. In the situation that a feature has missing values because its abundance is below the limit of detection of the instrument used, there is a possibility that it is a hormone/signaling molecule and, as such, may hold great promise as a disease biomarker. These metabolites could potentially be identified by a more sensitive targeted analysis, but, unfortunately, such potentially useful biomarkers will often suffer from the masking effects of imputation at the untargeted stage, due to their high levels of missingness, meaning that their signal is obliterated at the early stage of the discovery pipeline. As such, these features may not end up in the list of leads to progress to the targeted stage and, therefore, may not get an opportunity to be assessed by the more sensitive targeted methods.

The options for dealing with missing values include eliminating those features with missing values from further analysis, ignoring those missing values during analysis, or dealing with the missing values by imputation methods. Imputation algorithms can be broadly categorised into single value methods, local methods, global methods, hybrid methods, and knowledge based methods [44,45,46]. A variety of imputation algorithms exist and have been evaluated and compared elsewhere [35,47,48] and it is acknowledged that no single imputation method is universally superior [44,49]. 

For a heterogeneous disease dataset with hidden subgroups, where a disease related feature is likely only dysregulated in a subset of cases, the issue of missing value imputation becomes delicate. Local, global or hybrid methods are likely to be unsuitable due to the presence of an unknown substructure in the data and also due to low sample numbers typical of most metabolomics studies, which would prevent correlation and covariance structures from being extracted reliably [50]. The imputation method employed for such a dataset should ideally aim to ensure that masking does not occur of a subtle signal from a feature that could potentially act as a biomarker for a subgroup of the disease.

The advice is to first assess if there is a need to impute [35]. Perhaps use of data analysis techniques that do not require an imputed dataset may fare best for CHD datasets. As has been stated previously, “any imputation method leads to bias”. An expectation of any method of imputation leading to an approximation of a faithful representation of the original missing values may be exceptionally unrealistic in the case of heterogeneous disease datasets. Instead, acceptance of the limitation that in the case of CHD datasets, that imputation methods will lead to even greater bias and that resulting imputed datasets are an approximation or artificial dataset. If, for the data analysis method being used, a complete dataset is essential, then the bias that is being imputed into the dataset should be factored into the data analysis. Also, post analysis and, if possible, investigation of identified differential features, without the imputed values, is advised.

## 5. The Data Analysis Step: Considerations for Complex Disease

### 5.1. The Reproducibility Crisis in Metabolomics Biomarker Discovery

The reproducibility crisis has incited considerable discussion in recent years [51,52], since the phrase first came to the fore when a major replication effort in psychology found that only 39% of studies assessed were reproducible. Since then, similar replication efforts have shown that the reproducibility crisis is a serious issue across a variety of disciplines, from cancer biology [53] to economics [54]. A 2016 survey by nature magazine showed that most scientific fields are facing a reproducibility crisis [55].

The terms reproducibility, replicibality, reliability, robustness, and generalizability all have slightly different meanings and implications and the study in [56] can provide clarification. In this review, results reproducibility and the related phenomenon of generalizability in metabolomics biomarker discovery are discussed.

With regards to metabolomics biomarker discovery, the issue of results irreproducibility can be described as the inability of results from preliminary analyses to reproduce in validation studies despite good performance on cross-validation/permutation on the initial dataset. In the field of metabolomics biomarker discovery, the majority of preliminary findings are not followed up by external validation [21]. The reason for this lack of follow up could be because validation studies do not actually take place or because validation studies are carried out and produce negative results, which are not reported (or published). A recent systematic review examined biomarker discovery publications where metabolomics biomarkers had been validated on an external test set (usually by the same group, on the same publication) and showed that apparently equivalent studies, on the same disease, obtain different biomarker lists [30]. 

The most often purported reasons in the literature for the reproducibility crisis in preclinical research are lack of standards and rigor in experimental procedures, publication bias, poor data analysis techniques, questionable research methods, selective reporting of results, studies with low statistical power, and a “faulty incentive” culture in scientific and clinical research [52,57]. Solutions emanating from this view of the crisis focus on open science practices, data sharing, and increased standardisation.

The above reasons for the reproducibility crisis notwithstanding, an alternative perspective on the reproducibility crisis has begun to appear in the literature, which suggests that expectations of reproducibility are misplaced. This view proposes that a large part of the reproducibility crisis in clinical and preclinical research can be ascribed to a failure to account for contextual sensitivity [58], phenotypic plasticity [59], and reaction norms [60]. This philosophy urges us to adjust our expectations [61] and, therefore, our analysis methods. Voekl [62] shows that increasing variability, rather than reducing it, improves reproducibility in preclinical animal studies. An [63] describes his perception of the reproducibility crisis as a failure of typical methods of scientific investigation to account for biological heterogeneity. Describing the denominator subspace as all possible states of a biosystem, An posits that typical experiments only base models on a sliver of possible outcomes and, as such, failure of these models to be generalizable (reproducible) is inevitable. 

**Figure 1:**
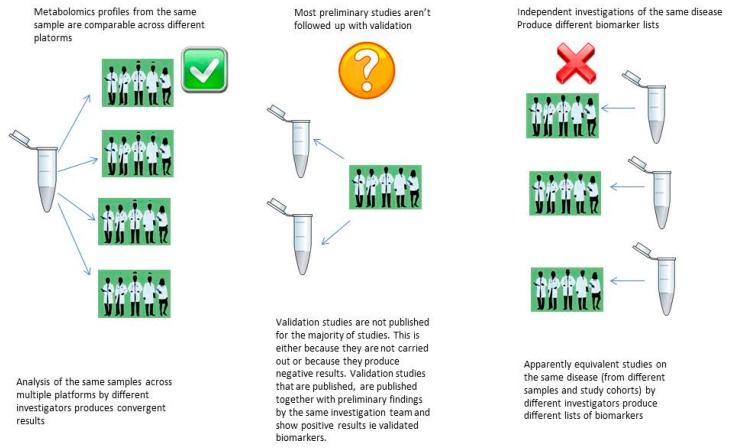
Can results irreproducibility in metabolomics be explained by contextual sensitivity?

In metabolomics biomarker discovery, as has been mentioned previously, validation efforts of preliminary discovery findings are in the vast minority (and are almost always carried out by the same group), and validated studies by different groups on the same disease produce different lists of biomarkers [15]. However, metabolomics profiles from the same samples analysed across different platforms, by different investigators, have produced comparable profiles even without prior standardisation [64]. Together, these findings could point towards contextual sensitivity as a possible explanation for results irreproducibility (Figure 1), as opposed to issues related to in experimental standards.

### 5.2. Multivariate Analysis and Univariate Analysis

Univariate and multivariate analysis techniques are frequently applied to metabolomics datasets and are considered to provide complementary information. Therefore, it is advised that both analysis methods be employed. Univariate techniques examine only one variable at a time while multivariate techniques make use of co-variances or correlations, which reflect the extent of the relationships among the variables. Based on the intuitive notion of gene-sets or metabolic networks, there is a general belief that multivariate analysis is superior to univariate analysis for the discovery of biomarker candidates [65].

However, Lai et al. show that univariate selection approaches yield generally better results than multivariate approaches across the majority of gene expression datasets that they analysed [50]. The reason for this, they conclude, is that correlation structures, even if they are present, cannot be extracted reliably due to low sample numbers. Another study shows that for gene extraction, multivariate statistics do not lead to a substantial gain in power compared with univariate statistics, even when correlations are present and high [66].

Multivariate search for biomarkers using supervised algorithms is based on the underlying assumption of homogeneity with multiple discriminating features together representing the fingerprint of the disease phenotype. However, in the situation where a CHD does not contain a hidden fingerprint for disease that is common to most or all cases, but instead exhibits perturbations in some features, in some cases, that reflect the different etiologies of the hidden subgroups, then the biomarker(s) from these different subsets of the disease are difficult to reveal using global methods due to their low overall prevalence among cases in the dataset [28].

Multivariate techniques, particularly statistical learning techniques, are the *de facto* data analysis methods employed for biomarker discovery in metabolomics. Instability and abstraction have been purported as the two fundamental issues contributing to the failure of biomarkers obtained from statistical learning approaches and are problems that are “here to stay” due to characteristics inherent in omics datasets. Instability is due to the curse of dimensionality, and the “small *n* large *p*” problem. Abstraction describes the data driven nature of the algorithms, which yield complex decision rules that generally lack meaning for biologists and clinicians to “generate testable hypotheses” [38]. Partial Least Squares Discriminant Analysis (PLSDA) is the most popular learning algorithm used in metabolomics biomarker discovery. Its widespread availability in omics analysis software is credited (or blamed) for the algorithm’s ubiquity in metabolomics biomarker discovery studies [67]. PLSDA, however, has been described as an “algorithm full of dangers” and is prone to overfitting and, therefore, producing false positive results [67,68,69]. PLSDA is also susceptible to misuse in the hands of non-experts [67]. For example, supervised algorithms such as PLSDA can result in biased estimations of prediction accuracy when classifiers are used that fail to select features from scratch in each iteration of a loop in cross-validation. This analysis flaw was found to be common in a review of microarray statistical analyses [70]. It is reasonable to assume that such misuse of PLSDA and similar algorithms is also an issue in metabolomics data analysis. However, poor reporting makes assessing the extent of this problem difficult [36]. Finally, as an especially important point for the analysis of complex and heaterogeneous datasets, Eriksson et al. note that “*a necessary condition for PLSDA to work is that each class is tight and occupies a small and separate volume in X-Space... Moreover, when some of the classes are not homogeneous and spread significantly in X-space, the discriminant analysis does not work*” [71]. Clearly, this has serious implications for the use of PLSDA to analyse CHD datasets.

PLSDA and other classification algorithms may have most success in biomarker discovery for CHD in metabolomics in certain scenarios: (1). If the dataset under study represents a subtype of the complex disease, so it is essentially homogeneous. (2). If, at the time of bio-specimen sampling, etiological pathways had converged sufficiently, so the samples under analysis were exhibiting the same pathway, so again, the dataset would essentially be homogeneous at that time point. However the first scenario is unlikely to exist the phenotypic classification does not, in fact, represent a biologically unique category but instead a lumping together of cases due to similar phenotypes, and the second scenario is unlikely to exist if the sample timing captures the metabolomic activity at different stages along the various etiological pathways of the disease or too early in the disease etiology at a point in time where the pathways have not yet converged. 

While multivariate modelling methods, such as PLSDA, often identify discriminating variables that lead to good classification upon cross validation and permutation, these variables are unlikely to be generalizable beyond the original system due to the complexity of the system from which they are derived. The discriminating variables identified in this scenario are likely to be highly context specific and might, in fact, not even be a revelation of real world phenomena but may, instead, merely represent “a tautological relationship between a set of numbers” [72] derived from a static snapshot analysis of a dynamic system. 

An [63] eloquently defines what he calls The Denominator Problem in biomedical research as the intrinsic inability of reductionist experimental biology to effectively reflect system denominator space, where the denominator is defined as the population distribution of the total possible behaviour/state space, as described by whatever metrics chosen, that biosystem. He explains how the requirements of experimental biology serve to constrain the sampling of denominator space, which leads to extreme sensitivity to conditions and irreproducibility. The author proposes that increased complexity and sophistication in the form of multi-scale mathematical (MSM) and dynamic computational models, to account for the denominator subspace, will overcome this problem. Geman [38] also suggests that global mathematical models over network scale configurations of genomic states and molecular concentrations will overcome the failure of omics based predictors and signatures to translate to clinical use.

Somewhat conversely, Karpievitch et al. describe how a major issue affecting the success of biomarker discovery in metabolomics is that the long line from pre-processing to statistical inference in data analysis is costly in terms of loss of degrees of freedom and accumulation of bias and errors [34]. At every step, there is a loss of degrees of freedom as we “use up” information in the dataset. Variability introduced at each step is not communicated to the next step, which can lead to an over-fitting of the data. Karpievitch et al. [34] suggest that the most natural solution to this problem is that processing and inference are ideally carried out in the same step, as this will greatly increase the quality of results by limiting the amount of bias communicated from one step to another. If this approach is not achievable, then shortening the data analysis pipeline, where possible, will result in fewer decisions to be made and, consequently, less opportunity for the introduction of bias, thereby increasing the possibility of obtaining actual meaningful results. 

**Figure 2:**
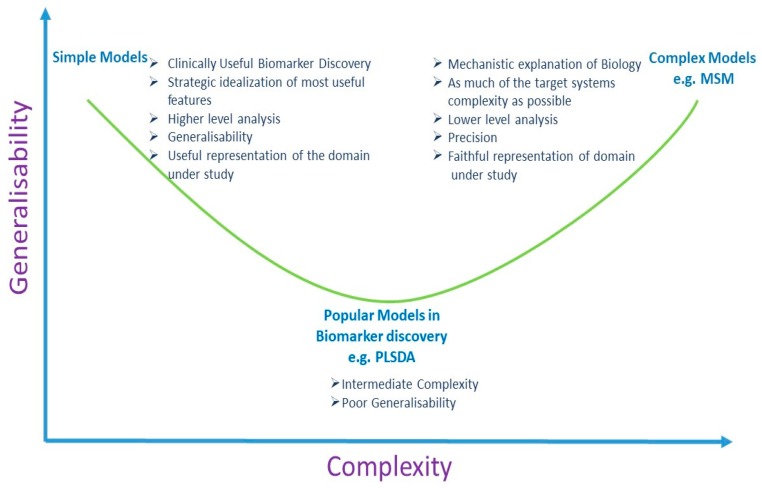
Simple models for biomarker discovery may be more generalisable. (PLSDA: Partial Least Squares Discriminant Analysis; MSM: Multi Scale Mathematical Models).

Therefore, it may be the case that typical data analysis techniques employed in biomarker discovery are at once too complex and not complex enough to tackle the biological heterogeneity issue. Specifically, statistical learning techniques, such as PLSDA, may be inadequate to model the heterogeneity and complexity of the “denominator subspace”, and, at the same time, they may be overly complex, leading to loss of information from an already overtaxed source. The trade-off of loss of degrees of freedom may not be balanced adequately by a gain in information. Until such a time as the sophistication of the methods improves (i.e., until multi-scale-modelling can be achieved), recognition of the limitations of what we can access from a static representation of a dynamic system needs to be accepted. While not sophisticated, low constraint and low complexity analysis methods, to search for biomarkers, may demonstrate improvements in generalisability. Figure 2 represents a schematic of this proposed notion. 

As an example, in the investigation of clinically useful biomarkers from a CHD metabolomics dataset, features that exhibit stable expression across all controls are a rich source of clinically applicable information. In a CHD dataset, the case group has hidden substructure due to unknown hidden subgroups of disease, whereas in the controls, at least for the outcome of interest, that hidden substructure does not exist. A feature that exhibits low variance or stable expression across a group of healthy controls and perturbation, in at least some cases, is likely to be informative, and, more importantly, that information is likely to be generalizable beyond the study at hand, since the feature has shown stability across a group of patients (the controls) and, as such, has demonstrated that it is impervious to context sensitivity (in the actual study at least). This would be a mechanism would simultaneously leverage prior information into model building that would constrain model complexity and overcome the issue of contextual sensitivity.

## 6. Discussion 

“Formal Analysis freezes moments of a process into things” [73]. When Richard Levins wrote this statement in his essay, “Formal analysis and the fluidity of science”, although he was not referring specifically to metabolomics analysis, it is very apt. Metabolomics is considered the most complex omics domains to model owing to its dynamic nature, which consists of non-linear relations (that are not fully understood); its sensitivity to environmental fluxes; and its stochasticity. In metabolomics biomarker discovery, analysis is carried out on a static, context specific representation of an extremely dynamic system. Indeed, metabolomics is frequently described as a “snapshot” analysis. This snapshot then undergoes a long line of processing to inference, using a complex model, with the aim of generalizability to other static representations of complex domains. 

The claim that multivariate approaches are more desirable than univariate selection processes for biomarker discovery was assessed for microarray datasets, and univariate methods were found to yield consistently better results than multivariate approaches [50]. A fair assessment of this claim has not yet been made for metabolomics studies. While the understanding of the molecular architecture of complex disease has undergone an awakening over the last decade in light of GWAS results, the de facto multivariate data analysis methods for complex disease biomarker discovery (which established their popularity with the original understanding of complex diseases as arising from common polygenic variants) have remained largely unchanged. It can be assumed that the early optimism in the superiority of supervised classification techniques in the search for biomarker signatures for complex diseases was due to an under-appreciation of the complexity of the genome and, hence, of the heterogeneity of complex diseases at the molecular level. 

The path to biomarker discovery has been predicted to be tortuous [49]. In data analysis, a variety of risk factors exist that contribute to the irreproducibility of results. Some of these risk factors can be addressed, while others are more difficult to address (but can, at least, be acknowledged). The risk factors inherent in metabolomics biomarker discovery can be compounded by employing data driven approaches and applying imputation methods without considering the potential loss of information. Heterogeneity of disease and datasets need to be accounted for via a more study specific and disease specific approach as opposed to an indiscriminate, one size fits all, “big data” approach [31]. That metabolomics ultimately holds the key to personalised medicine is not in question in this perspective. However, a pragmatic approach to biomarker discovery is required, where the clinical need, as well as the available biological knowledge and understanding of the molecular basis of the disease under study (and its etiology), inform all aspects of the early discovery process, from the design of experiment and the design of the biomarker discovery pipeline, to the choice of data pretreatment and data analysis strategies.

The simplicity principle (also known as Occam’s Razor) tells us that more complex theories, due to their complexity and flexibility, are more likely to fit more closely to the data, but simpler theories will generalise better. The main aim of clinically applicable biomarker discovery is their subsequent generalisability as opposed to their predictive performance in preliminary studies. In modelling, any one of the three attributes (precision, realism, or generalisability) can be maximised at the expense of the other two attributes [73]. For the purpose of biomarker discovery for clinical application, the generalisability of the model, rather than its precision or realism, is the most important aspect. Alternatively, when an understanding of a biological system is sought, then the importance of precision and realism supersedes generalisability. The discovery of biomarkers for clinical diagnosis and prognosis is urgently required. In the intervening time until sophisticated analyses, like multi scale methods, are implemented, simpler models may have more success in identifying generalizable biomarkers.
